# A randomized trial comparing methotrexate and vinblastine (MV) with cisplatin, methotrexate and vinblastine (CMV) in advanced transitional cell carcinoma: results and a report on prognostic factors in a Medical Research Council study. MRC Advanced Bladder Cancer Working Party.

**DOI:** 10.1038/bjc.1998.629

**Published:** 1998-10

**Authors:** G. M. Mead, M. Russell, P. Clark, S. J. Harland, P. G. Harper, R. Cowan, J. T. Roberts, B. M. Uscinska, G. O. Griffiths, M. K. Parmar

**Affiliations:** Royal South Hants Hospital, Brintons Terrace, Southampton, UK.

## Abstract

Transitional cell carcinomas may arise at any site within the urinary tract and are a source of considerable morbidity and mortality. In particular, patients with metastatic disease have a poor prognosis, with less than 5% alive at 5 years. A multicentre randomized trial comparing methotrexate and vinblastine (MV) with cisplatin, methotrexate and vinblastine (CMV) in advanced or metastatic transitional cell carcinoma was conducted in the UK. From April 1991 to June 1995, 214 patients were entered by 16 centres, 108 randomized to CMV and 106 to MV. A total of 204 patients have died. The hazard ratio (relative risk of dying) was 0.68 (95% CI 0.51-0.90, P-value = 0.0065) in favour of CMV. This translates to an absolute improvement in 1-year survival of 13%, 16% in MV and 29% in CMV. The median survival for CMV and MV was 7 months and 4.5 months respectively. Two hundred and eight patients objectively progressed or died. The hazard ratio was 0.55 (95% CI 0.41-0.73, P-value = 0.0001) in favour of CMV. Two hundred and nine patients symptomatically progressed or died. The hazard ratio was 0.48 (95% CI 0.36-0.64, P-value = 0.0001) in favour of CMV. The most important pretreatment factors influencing overall survival were WHO performance status and extent of disease. These two factors were used to derive a prognostic index which could be used to categorize patients into three prognostic groups. We conclude that the addition of cisplatin to methotrexate and vinblastine should be considered in patients with transitional cell carcinoma, taking into account the increased toxicity.


British Journal of Cancer (1998) 78(8). 1067–1075
© 1998 Cancer Research Campaign

### A randomized trial comparing methotrexate and vinblastine (MV) with cisplatin, methotrexate and vinblastine (CMV) in advanced transitional cell carcinoma: results and a report on prognostic factors in a Medical Research Council study

**GM Mead[1], M Russell[2], P Clark[3], SJ Harland[4], PG Harper[5], R Cowan[6], JT Roberts[7], BM Uscinska[8], GO Griffiths[8] and MKB Parmar[8] on behalf of the MRC Advanced Bladder Cancer Working Party**

[1]Royal South Hants Hospital. Brintons Terrace. Southampton SO14 0YG. UK: [2]Beatson Oncology Centre. Western Infirmary. Glasgow G11 6NT. UK: [3]Clatterbridge Centre for Oncology. Clatterbridge Road. Bebington. Wirral. Merseyside L63 4JY. UK: [4]University College and Middlesex School of Medicine. 48 Riding House Street. London W1P 7PN. UK: [5]Guy's Hospital. St Thomas Street. London SE1 9RT. UK: [6]Christie Hospital. Wilmslow Road. Manchester M20 9BX. UK: [7]Northern Centre for Cancer Treatment. Newcastle General Hospital. Westgate Road. Newcastle-Upon-Tyne NE4 6BE. UK: [8]MRC Cancer Trials Office. 5 Shaftesbury Road. Cambridge CB2 2BW. UK

**Summary** Transitional cell carcinomas may arise at any site within the urinary tract and are a source of considerable morbidity and mortality. In particular. patients with metastatic disease have a poor prognosis. with less than 5% alive at 5 years. A multicentre randomized trial comparing methotrexate and vinblastine (MV) with cisplatin. methotrexate and vinblastine (CMV) in advanced or metastatic transitional cell carcinoma was conducted in the UK. From April 1991 to June 1995. 214 patients were entered by 16 centres. 108 randomized to CMV and 106 to MV. A total of 204 patients have died. The hazard ratio (relative risk of dying) was 0.68 (95% CI 0.51–0.90. *P*-value = 0.0065) in favour of CMV. This translates to an absolute improvement in 1-year survival of 13%. 16% in MV and 29% in CMV. The median survival for CMV and MV was 7 months and 4.5 months respectively. Two hundred and eight patients objectively progressed or died. The hazard ratio was 0.55 (95% CI 0.41–0.73. *P*-value = 0.0001) in favour of CMV. Two hundred and nine patients symptomatically progressed or died. The hazard ratio was 0.48 (95% CI 0.36–0.64. *P*-value = 0.0001) in favour of CMV. The most important pretreatment factors influencing overall survival were WHO performance status and extent of disease. These two factors were used to derive a prognostic index which could be used to categorize patients into three prognostic groups. We conclude that the addition of cisplatin to methotrexate and vinblastine should be considered in patients with transitional cell carcinoma. taking into account the increased toxicity.

**Keywords:** chemotherapy: transitional cell carcinoma: randomized

Transitional cell carcinomas (TCCs) may arise at any site within the urinary tract and are a source of considerable morbidity and mortality. In 1994. there were 5300 deaths from this disease in the UK (Cancer Research Campaign. 1995). Approximately 90% of these cancers arise in the bladder. occurring at a median age of 65 years. Treatment of patients with disease confined to the bladder (T2 or T3) with radiotherapy or cystectomy results in cure in 30–40% of cases. Patients with metastatic disease have a much poorer prognosis. with less than 5% alive at 5 years (Saxman et al. 1997).

During the last 15–20 years. chemotherapy. predominantly using the drugs methotrexate. vinblastine. cisplatin and doxorubicin. has been widely used to treat these cancers (Sternberg. 1995). Early randomized trials using combinations of these drugs. usually compared with cisplatin as a single-agent control. were able to demonstrate their moderate activity (Gagliano et al. 1983: Soloway et al. 1983: Khandekar et al. 1985: Troner et al. 1987: Hillcoat et al. 1989). However. response and survival were generally short and no clear benefit for combination chemotherapy could be demonstrated. However. with the development of methotrexate vinblastine adriamycin(doxorubicin) cisplatin (M-VAC) (Sternberg et al 1985. 1988. 1989). a drug combination incorporating all these drugs. and CMV (omitting doxorubicin: Harker et al. 1985: Jeffery et al. 1992). large improvements in remission rate were reported in single-institution studies. with reports of long-term survival in 20% of patients in one study (Sternberg et al. 1989). In a subsequent randomized trial. however. comparing single-agent cisplatin with M-VAC (Loehrer et al. 1992). the essentially palliative nature of these treatments was demonstrated. While M-VAC proved capable of increasing median survival time from 8 months to 12 months. a 5-year progression-free survival of only 4% was reported (Saxman et al. 1997). Similarly. in a large retrospective study of patients receiving cisplatin combination chemotherapy for locally advanced or metastatic urothelial cancer (Fossa et al. 1996). a 5-year survival rate of only 11% was reported. M-VAC has also been reported to improve survival when compared with CISCA (cisplatin. cyclophosphamide and doxorubicin) in a randomized trial (Logothetis et al. 1990).

*Received 3 December 1997*
*Revised 3 March 1998*
*Accepted 17 March 1998*

*Correspondence to:* MKB Parmar

Combination chemotherapy including cisplatin is toxic, resulting in marked morbidity for the majority of patients and treatment-related mortality in up to 4% of cases (Loehrer et al, 1992). Cisplatin itself is probably responsible for most of the impairment of quality of life, and the precise role of this drug in the management of these cancers has not been clearly demonstrated. Cisplatin-based treatment can also be inconvenient to the patient, usually requiring hospitalization for administration. In the trial comparing single-agent cisplatin with M-VAC (Loehrer et al, 1992), an overall response rate of only 12% was described for cisplatin used as a single agent.

In 1991, the British Medical Research Council initiated a trial comparing combination chemotherapy with CMV (cisplatin given at a dose of 70 mg $m^{-2}$) with a modified methotrexate and vinblastine (MV) regimen. This latter regimen can be given on an outpatient basis and was reported as providing a 40% response rate (complete response plus partial response) in metastatic transitional cell cancer when used on a weekly basis (Ahmed et al, 1985). This trial was designed to evaluate the impact of cisplatin on this disease.

### PATIENTS AND METHODS

### Study design and randomization

Patients eligible for this study had to have a histologically confirmed diagnosis of transitional cell carcinoma arising at any site in the urothelial tracts. Patients with mixed tumours (i.e. tumours containing elements of squamous cell or adenocarcinoma) were also eligible for inclusion in this study, although patients with pure non-TCC tumours were excluded. Patients should have been considered incurable by surgery or radiotherapy and the following groups were included: (1) metastatic disease at any site (including completely resected pelvic nodal disease), (2) invasive pelvic relapse after radical radiotherapy and (3) initial presentation with T4b disease. It was not considered essential that patients had measurable disease, as the primary end point of the study was length of survival.

Further eligibility criteria were as follows: all patients were required to have a normal blood count (WBC > $3.5 \times 10^9$ $l^{-1}$ with a platelet count > $100 \times 10^9$ $l^{-1}$) and a glomerular filtration rate (GFR), calculated by the method of Cockcroft and Gault (1976) of > 50 ml $min^{-1}$, if necessary achieved by ureteric stenting or percutaneous drainage of the urinary tract where obstruction was present. All patients had to be considered fit to withstand treatment with cisplatin-containing chemotherapy, and no previous systemic chemotherapy was permitted. Patients with concomitant or previous malignancy other than basal cell carcinoma of the skin or CIS of the cervix were also excluded from study entry. The protocol was reviewed in each institution by the local ethics committees, and informed consent to inclusion in the study was given by all patients.

Eligibility of patients was confirmed and randomization performed by a telephone call to the Medical Research Council Cancer Trials Office. Randomization was by the method of minimization with stratification factors of centre, performance status and the presence or absence of visceral disease.

To help design this trial, members of the Advanced Bladder Cancer Working Party were asked what, in their opinion, was the improvement in 2-year survival they would wish to see before changing treatment from MV to CMV. The overall results indicated that approximate clinical equivalence would be demonstrated if the absolute benefit to CMV was less than 10–15%. To exclude an absolute improvement larger than 15% (that is, say, from 20% in the CMV arm to 5% or less in the MV arm) required that 200 patients were randomized (significance level = 10%, power = 90%). Randomization of 400 patients would allow us to exclude a difference of 10% with the same significance level and power. It was decided to aim for 200 patients in the first instance and, if adequate accrual was attained, the trial would continue to enter 400 patients.

### Treatments

All patients were planned to receive six cycles of either MV or CMV. Patients were re-evaluated after two treatment cycles; if treatment-related symptoms were stable, or improved and simple re-evaluation (physical examination, liver function tests, chest radiography and abdominal and/or pelvic ultrasound) showed no evidence of disease progression, treatment was continued, in the absence of disease progression, for six cycles.

Both regimens, MV and CMV, were given over a 21-day cycle. MV comprised methotrexate at 30 mg $m^{-2}$ given by slow intravenous push on days 1 and 8 and vinblastine at 4 mg $m^{-2}$ given by intravenous push on days 1 and 8. Folinic acid rescue was given 24 h after each methotrexate injection at a dose of 15 mg orally, 6-hourly × 4. CMV comprised MV given exactly as described, but included, in addition, inpatient administration of cisplatin at a dose of 70 mg $m^{-2}$ on day 2. Cisplatin was given following a period of i.v. hydration in which at least 2 l of normal saline was given, and was not administered until urine output was measured as equalling or exceeding 100 ml $h^{-1}$ for 4 h. Cisplatin was administered in 500 ml of normal saline over 1 h and was followed by at least 2 l further hydration with normal saline, with supplementary potassium chloride and magnesium sulphate.

All three chemotherapy drugs were given at full dose, on time, if the white blood count was > $3.5 \times 10^9$ $l^{-1}$ with a platelet count of > $100 \times 10^9$ $l^{-1}$ and calculated GFR was > 50 ml $min^{-1}$. Methotrexate and vinblastine doses were reduced by 25% for WBC 3–$3.5 \times 10^9$ $l^{-1}$ and by 50% for WBC 2.5–$2.9 \times 10^9$ $l^{-1}$. A WBC of < $2.5 \times 10^9$ $l^{-1}$ or platelets < $100 \times 10^9$ $l^{-1}$ on day 1 caused delay of chemotherapy by up to 2 weeks; on day 8, chemotherapy was omitted if these counts were found. A GFR of 35–50 ml on day 2 resulted in a reduction of cisplatin dose by 50%. Methotrexate and cisplatin were omitted if the GFR was < 35 ml $min.^{-1}$

### Investigations before and during treatment

Before entry into the study, a full physical examination was performed and the WHO performance status recorded. A full blood count and biochemical profile (including liver function tests, electrolytes and urea and creatinine) were performed together with chest radiography. CT scans of the chest, abdomen and pelvis were obtained as clinically indicated. Bone scanning was not mandatory, but rather directed by symptoms.

Before each course of chemotherapy on days 1 and 8, a full blood count and serum creatinine were obtained. At the end of chemotherapy, formal re-evaluation was performed, repeating all initially abnormal investigations (except bone scanning) found at the initiation of treatment.

### End points and analysis

The date of first progression of cancer-related symptoms, first date of objective disease progression (found on physical examination

 © Cancer Research Campaign 1998

or radiologically) and overall survival were measured from the date of randomization. Survival and progression-free survival curves were formed by the Kaplan–Meier method and compared using the Mantel–Cox version of the log-rank test. To assess whether CMV or MV were more or less effective in well-defined subgroups, a $\chi^2$ test for heterogeneity or, when appropriate, trend was performed. All analyses were performed on an intention-to-treat basis, all tests are from a $\chi^2$ distribution with one degree of freedom and all *P*-values are two-sided unless otherwise specified (Parmar and Machin, 1995). The statistical methods used were implemented using SAS (1989).

Absolute benefits at specific time points for CMV for overall survival were calculated using the Kaplan–Meier estimate for survival on the MV arm at that time point (baseline survival), using the expression: absolute benefit = exp (hazard ratio × log baseline) – baseline survival. This approach was also adopted for the end points of objective and symptomatic progression-free survival. Although this approach implicitly assumes proportional hazards, it is preferable to reading off differences between the Kaplan–Meier curves at individual time points (Parmar and Machin, 1995).

Where possible, tumour response was recorded as the best response achieved during chemotherapy. Bone disease was regarded as non-evaluable. Complete remission required total disappearance of disease both on physical examination and radiologically. Partial remission was defined as a reduction of at least 50% in the sum of the product of the cross-sectional diameters of all measurable lesions, without progression at any site. Progressive disease was defined as a > 25% increase recorded in the size of any lesion. If patients did not satisfy any of these criteria, they were defined as having stable disease.

Analysis of prognostic factors was done by using the Cox proportional hazards model. To build a model, univariate analyses were done using a *P*-value of 0.10 to determine whether to include a variable in the overall model. A forward selection procedure was used to build a model and a prognostic index was developed. The methods used in this whole process are described in Parmar and Machin (1995).

### RESULTS

From April 1991 to June 1995, 214 patients were entered into this multi-institution study from a total of 16 centres within the UK. Entry by institution is shown in Table 1 and patient characteristics are shown in Table 2. The patients were well matched with regard to these patient characteristics in the two treatment groups.

### Treatment delivery and response

One hundred and eight patients were randomized to receive combination chemotherapy with CMV and 106 patients were randomized to receive MV chemotherapy (Table 3). Forty patients (37%) completed a total of six cycles of CMV treatment – the median number of cycles received was four. Twenty-two patients (21%) allocated MV completed six cycles of treatment and the median number of cycles received was three. One patient in the MV arm changed his mind after randomization and opted for CMV chemotherapy.

Disease progression occurred during chemotherapy in 34 patients (32%) receiving CMV and 72 patients (68%) receiving MV. Clinical response was not a primary end point of this study.

**Figure 1** Kaplan–Meier curves of objective progression-free survival in the two treatment groups

**Figure 2** Kaplan–Meier curves of symptomatic progression-free survival in the two treatment groups

However, of 88 patients allocated CMV with evaluable disease, a complete response (CR) occurred in 10% with a partial response (PR) in 36% (CR + PR = 46%). Among 93 evaluable patients allocated MV, a complete response occurred in 7% with a partial response in 12% (CR + PR = 19%).

### Objective progression-free survival

A total of 208 patients have objectively progressed or died, 104 allocated CMV and 104 allocated MV. A comparison of the Kaplan–Meier curves (Figure 1) for the two treatments gives a hazard ratio of 0.55 (*P*-value = 0.0001; 95% confidence interval = 0.41–0.73), indicating a 45% reduction in the relative risk of progression or death with CMV when compared with MV. This

© Cancer Research Campaign 1998

**Table 1** Number of patients entered by each centre

| Centre | Total |
|---|---|
| Airedale General Hospital | 4 |
| Beatson Oncology Centre/Belvidere Hospital, Glasgow | 39 |
| Bristol Oncology Centre | 11 |
| Cheltenham General | 3 |
| Christie Hospital, Manchester | 17 |
| City/Queen Elizabeth Hospital, Birmingham | 9 |
| Clatterbridge Centre for Oncology | 21 |
| Cookridge Hospital, Leeds | 2 |
| Guys Hospital, London | 18 |
| Middlesex Hospital, London | 20 |
| Newcastle General/Freeman Hospital, Newcastle | 22 |
| Royal Free Hospital, London | 1 |
| Royal South Hants/St Marys Hospital, Southamptom | 28 |
| Velindre Hospital, Cardiff | 3 |
| Westmorland General Hospital/Royal Lancaster Infirmary | 8 |
| Weston Park Hospital, Sheffield | 8 |
| Total | 214 |

translates to an improvement in median objective progression-free survival of 2.5 months (from 3 months to 5.5 months).

### Symptomatic progression-free survival

A total of 209 patients have experienced symptomatic progression or died, 104 allocated CMV and 105 allocated MV. The Kaplan–Meier curves for the two treatments are shown in Figure 2. Comparing these two curves gives a hazard ratio of 0.48 ($P$-value = 0.0001; 95% confidence interval = 0.36–0.64), indicating a 52% reduction in the relative risk of symptomatic progression or death with CMV. This translates to a 2.5 month improvement in median symptomatic progression-free survival (from 2 months to 4.5 months).

### Overall survival

A total of 204 patients have died, 101 allocated CMV and 103 allocated MV. A comparison of the Kaplan–Meier curves (Figure 3) gives a hazard ratio of 0.68 ($P$-value = 0.0065; 95% confidence interval = 0.51–0.90), indicating a 32% reduction in the relative risk of death with CMV. This translates to a 2.5-month improvement in median survival (from 4.5 months to 7 months) and an absolute improvement of 13% in 1-year survival (from 16% to 29%).

At the time of analysis, seven patients allocated CMV remain alive, four with disease and three without. The three patients without disease had no further treatment. Two of the patients with disease had further treatment – one cystectomy and one MVAC. Of those allocated MV, three patients remain alive, one with disease and two without. Of the two patients without disease, both have had further treatment: one has had radiotherapy and chemotherapy and the other has had a cystectomy. The one patient alive with disease has had no further treatment for bladder cancer but has had treatment for prostate cancer.

### Toxicity

CMV treatment was associated with considerably more toxicity than MV. A total of five treatment-related deaths occurred in patients receiving CMV (4%) and none in patients receiving MV.

**Table 2** Patient characteristics

| | Treatment allocated | | |
|---|---|---|---|
| | CMV (%) | MV (%) | Total (%) |
| Age (years) | | | |
| ≤ 65 | 60 (56) | 66 (62) | 126 (59) |
| > 65 | 48 (44) | 40 (38) | 88 (41) |
| Median | 65 | 64 | 64 |
| Sex | | | |
| Male | 83 (77) | 83 (78) | 166 (78) |
| Female | 25 (23) | 23 (22) | 48 (22) |
| WHO performance status | | | |
| 0 | 30 (28) | 25 (23) | 55 (26) |
| 1 | 51 (47) | 53 (50) | 104 (48) |
| 2 | 21 (19) | 20 (19) | 41 (19) |
| 3 | 6 (6) | 8 (8) | 14 (7) |
| Time since presentation (months) | | | |
| 0–5 | 60 (55) | 46 (44) | 106 (49) |
| 6–12 | 18 (17) | 29 (27) | 47 (22) |
| > 12 | 30 (28) | 31 (29) | 61 (29) |
| Site of primary tumour | | | |
| Bladder | 96 (89) | 95 (90) | 191 (89) |
| Other (kidney, prostate, ureter) | 12 (11) | 11 (10) | 23 (11) |
| Previous treatment | | | |
| None | 31 (29) | 24 (23) | 55 (26) |
| Surgery | 31 (29) | 22 (21) | 53 (25) |
| Radiotherapy ± surgery | 46 (42) | 60 (56) | 106 (49) |
| Extent of disease | | | |
| Visceral (bone, liver, lung, other) | 61 (56) | 56 (53) | 117 (55) |
| Nodal (pelvis/abdominal) | 39 (36) | 37 (35) | 76 (35) |
| Bladder relapse | 4 (4) | 7 (7) | 11 (5) |
| T4b at presentation | 4 (4) | 6 (6) | 10 (5) |
| Total | 108 (100) | 106 (100) | 214 (100) |

**Table 3** Summary of treatment

| | Treatment allocated | | |
|---|---|---|---|
| | CMV (%) | MV (%) | Total (%) |
| Treatment completed (six cycles) | 40 (37) | 22 (21) | 62 (29) |
| Disease progression[a] | 34 (32) | 72 (68) | 106 (50) |
| Toxic death during treatment | 5 (4) | 0 | 5 (3) |
| Excessive toxicity – treatment stopped | 16 (15) | 0 | 16 (7) |
| Intercurrent death (due to neither toxicity nor progression) | 2 (2) | 2 (2) | 4 (2) |
| Other medical condition – treatment stopped[b] | 5 (4) | 4 (4) | 9 (4) |
| Treatment stopped by clinician because no improvement observed | 3 (3) | 5 (4) | 8 (3) |
| Treatment refusal | 3 (3) | 1 (1) | 4 (2) |
| Total | 108 (100) | 106 (100) | 214 (100) |

[a]Six patients (three CMV, three MV) progressed before starting chemotherapy and received no chemotherapy at all. [b]1 patient on CMV did not start chemotherapy because of cardiac problems.

The cause of death in these cases was cardiovascular toxicity (two patients), septicaemia (two patients) and renal failure (one patient). A further 16 patients (15%) receiving CMV were unable to complete this treatment because of excessive toxicity, and three more patients (3%) refused to continue this treatment, whereas, in patients receiving MV, no excessive toxicity problems were

© Cancer Research Campaign 1998

**Figure 3** Kaplan–Meier curves of overall survival in the two treatment groups reported and only one patient refused to continue treatment. CMV resulted in grade III leucopenia or thrombocytopenia in five cases vs no cases with MV. Neutropenic fever requiring hospital admission and intravenous antibiotics was recorded in 11 patients receiving CMV and two receiving MV. Grade I or II renal toxicity occurred in, respectively, 19 cases and four patients receiving CMV and MV.

Long-term toxicity (neurological) was reported in nine CMV patients and one patient on MV (although this patient actually received CMV).

### Effects in different subgroups

Table 4 shows the comparative effect of CMV and MV in different subgroups, for the main end point of overall survival. For each, the $\chi^2$ test for interaction is presented, or where appropriate the $\chi^2$ test for trend. There is some evidence of a larger effect in poor performance status patients as opposed to good-performance patients (Figure 4). There was no good evidence that the overall improved survival effect observed with CMV was larger or smaller in any of the other subgroups investigated.

### Prognostic factors

The seven characteristics (factors) of patients collected before randomization and treatment (and presented in Table 2) were analysed to assess whether they provided information which may help to predict the prognosis of patients. Initially, all the factors were analysed individually. The results of this analysis are presented in Table 5. Only two factors, WHO performance status and extent of disease, provide any good evidence of a relationship with overall survival. Kaplan–Meier survival curves for the three WHO performance status groups and the extent of disease groups are shown in Figures 5 and 6. For extent of disease, the groups of patients with nodal and bladder relapse/T4b disease had a similar prognosis and, thus, were combined. In further analyses, therefore, extent of disease was defined as visceral or non-visceral.

As WHO performance status was the factor with most evidence of a relationship with overall survival, the other six characteristics were added in turn to see if they contributed further information above and beyond this factor. The only one for which there was good evidence of adding information was extent of disease (chi-square for inclusion = 14.575 on 1 degree of freedom, *P*-value = 0.0001). In the next step, the remaining five factors were added to the model containing WHO performance status and extent of disease. There was no evidence for any of the five remaining factors adding further information.

Thus, from the seven factors considered, we conclude that only WHO performance status (0, 1, 2/3) and extent of disease (visceral, non-visceral) give useful independent information on the likely survival of patients. Table 6 shows the final Cox model with the estimates of the regression coefficients. To simplify the model, we attempted to develop a prognostic index. The prognostic index (PI) is used to derive a score from the key patient characteristics of WHO and extent of disease, which can then be used to indicate whether a patient has a good, intermediate or poor prognosis.

To derive a PI, it is usual to simplify the regression coefficients in the fitted Cox model. The exponent part of the fitted Cox model is 0.411W + 0.545E; preserving the ratio of the coefficients, 0.411:0.545 can be simplified to 3:4, giving PI = 3W + 4E (hence the index scores in Table 6).

The PI can be calculated for each patient, which gives a range from 0 to 10, a high score of PI indicating a poorer prognosis and a low score a better prognosis. The distribution of PI was examined and convenient subgroups of prognosis were identified. The good-prognosis group were defined as having a PI < 4; this group of patients includes those with WHO 0 or 1 and non-visceral disease.

**Table 4** Effects in different subgroups

| Subgroup | Categories | Chi-square value from test of interaction/trend | Degrees of freedom | *P*-value |
|---|---|---|---|---|
| Age | ≤65, > 65 | 0.342 | 1 | 0.559 |
| Sex | Male, female | 0.049 | 1 | 0.825 |
| WHO performance status | 0, 1, 2/3 | 5.395 | 1 | 0.020 |
| Time since presentation (months) | 0–5, 6–12, > 12 | 0.351 | 1 | 0.554 |
| Site of tumour | Bladder, kidney/prostate/ureter | 2.003 | 1 | 0.157 |
| Previous treatment | None, surgery, radiotherapy ± surgery | 1.409 | 2 | 0.494 |
| Extent of disease | Visceral, nodal, bladder relapse/T4b | 1.204 | 2 | 0.548 |

© Cancer Research Campaign 1998

**Table 5** Results of analysis of the relationship between pretreatment characteristics (factors) and survival

| Pretreatment characteristics | Chi-square value | Degrees of freedom | P-value |
|---|---|---|---|
| Age | | | |
| As a continuous variable | 0.813 | 1 | 0.367 |
| As a categorical variable (≤65, > 65) | 0.360 | 1 | 0.549 |
| Sex | | | |
| (male, female) | 0.317 | 1 | 0.573 |
| WHO performance status | | | |
| (0, 1, 2/3) | 17.244 | 1 | 0.00003 |
| Time since presentation (months) | | | |
| As a continuous variable | 0.024 | 1 | 0.877 |
| As an ordered categorical variable (0–5, 6–12, > 12) | 0.232 | 1 | 0.630 |
| Previous treatment | | | |
| (none, surgery, radiotherapy ± surgery) | 0.095 | 2 | 0.954 |
| Extent of disease | | | |
| (visceral, nodal, bladder relapse/T4b) | 16.486 | 2 | 0.0003 |
| (visceral/non-visceral) | 16.463 | 1 | 0.00005 |

**Table 6** Prognostic factors and prognostic index scores

| Prognostic factor | Category | Category score | Estimated coefficient | SE | HR | Index score |
|---|---|---|---|---|---|---|
| WHO (W) | 0 | 0 | 0 | – | 1 | 0 |
| | 1 | 1 | 0.411 | 0.105 | 1.508 | 3 |
| | 2/3 | 2 | 0.822 | – | 2.274 | 6 |
| Extent of disease (E) | Non-visceral | 0 | 0 | – | 1 | 0 |
| | Visceral | 1 | 0.545 | 0.144 | 1.725 | 4 |

(no. events/no. entered)

| | CMV | MV | O–E | Variance |
|---|---|---|---|---|
| WHO 0 | 28/30 | 22/25 | 0.30 | 12.23 |
| WHO 1 | 48/51 | 53/53 | −11.67 | 22.79 |
| WHO 2/3 | 25/27 | 28/28 | −10.30 | 10.99 |

**Figure 4** Hazard ratio plot of overall survival by WHO performance status

The intermediate-prognosis group had a PI of 4–6: this group includes those with either a WHO 2/3 and no visceral disease or WHO 0 and visceral disease. The poor-prognosis group had a PI > 6: this group includes those with WHO > 0 and visceral disease. Table 7 shows the number of patients in each of these groups with their median survival and 1-year survival rate. The survival curves for each of these risk groups are shown in Figure 7.

### DISCUSSION

Transitional cell carcinomas occur in a relatively elderly population in whom coexisting medical illnesses are common. In practice, chemotherapy is difficult to give to these patients – both because of the toxicity of the drugs at present in use and also because of the commonly poor performance status of these patients. A particular problem is impaired renal function, often caused by obstructive uropathy, which may preclude therapy with cisplatin and methotrexate. Many patients are not sufficiently fit to receive treatment with chemotherapy for this disease. Among those that are treated, it has become increasingly clear that treatment is palliative for all except a small subgroup. In particular, patients with visceral disease (particularly affecting the liver) are rarely, if ever, cured. However, patients with nodal disease or advanced pelvic disease at presentation (T4b), particularly those with a good performance status, may be cured and thus may warrant an intensive cisplatin-based treatment (Fossa et al, 1996).

© Cancer Research Campaign 1998

**Table 7** Number of patients, median and 1-year survival according to prognostic group

| Prognostic index | Risk group | Number of patients (%) | Median survival (days) | One-year survival |
|---|---|---|---|---|
| < 4 | Good | 77 (36) | 271 | 35% |
| 4–6 | Medium | 46 (21) | 221 | 24% |
| > 6 | Poor | 94 (43) | 112 | 15% |

**Figure 5** Kaplan–Meier curves of overall survival by WHO performance status

The results described in this, the second largest randomized trial reported in the literature, are inferior to those reported in both the recent large randomized studies, i.e. the Intergroup trial comparing cisplatin with M-VAC (Loehrer et al, 1994) and the MD Anderson study of M-VAC vs CISCA (Logothetis et al, 1990). There are a number of possible explanations for this. We deliberately chose to be as inclusive as possible in our study to represent as nearly as possible the true nature of this patient population. In this regard, we accepted patients with a relatively low GFR (greater than 50 ml $min^{-1}$) and used a lower dose of cisplatin than used in the original CMV regimen (70 mg $m^{-2}$ vs 100 mg $m^{-2}$; Harker et al, 1986), although there is no good evidence to suggest that this latter approach may be disadvantageous. Approximately 50% of our patients had received previous radiation compared with 25–30% in most series derived from the US. We deliberately included patients with non-measurable disease, a common situation following pelvic radiation which may be associated with an adverse outlook (Jeffery et al, 1992).

The study adequately highlighted the beneficial effect of cisplatin, increasing the median survival from 4.5 to 7.5 months and the 1-year survival from 16% to 29%. Improvements were also seen in symptomatic and objective progression-free survival. However, the toxicity of CMV and the relative inefficacy of both these regimens was highlighted both by the low proportion of patients completing the planned six cycles of chemotherapy and by the high proportion of patients discontinuing chemotherapy because of disease progression or excessive toxicity.

**Figure 6** Kaplan–Meier curves of overall survival by extent of disease

**Figure 7** Kaplan–Meier curves of overall survival for good-, medium- and poor-risk groups

A study by Saxman et al (1997) found that the best pretreatment predictors of survival in patients with metastatic urothelial carcinoma included performance status, histology and the presence of liver or bone metastases. In this study, using a population of patients with transitional cell carcinoma only, we found performance status and extent of disease to be the best predictors of survival. The extent of disease defined as visceral/non-visceral corresponds largely to the liver and bone metastases variables used by Saxman et al. We used the factors of performance status and extent of disease to produce a prognostic index, which could be used to classify patients into groups.

We, like other investigators (Tannock et al, 1989), conclude from this study that the drugs incorporated in CMV (or M-VAC), while achieving short-term disease regression, on the whole

*© Cancer Research Campaign 1998*

provide poor palliation for a majority of patients. A number of studies have attempted to increase the dose intensity of M-VAC by simultaneous administration of growth factors (Loehrer et al. 1994; Logothetis et al. 1985). The majority of these studies have concluded that this resulted in a modest increase in dose intensity, with markedly increased toxicity with no obvious clinical benefits. However, the European Organization for Research and Treatment of Cancer, following a successful randomized phase II trial in which no increase in toxicity was seen (Sternberg et al. 1997), is currently randomizing patients in a phase III trial between M-VAC and accelerated M-VAC supported by growth factors.

A number of new drugs and drug combinations are now under early stages of evaluation by ourselves and others. These agents include the taxanes, paclitaxel (Roth et al. 1994) and docetaxel (McCaffrey et al. 1995), gemcitabine (Stadler et al. 1995) ifosfamide (Witte et al. 1997) and gallium (Seligman et al. 1991). Other drugs such as fluorouracil are being re-evaluated and the MRC has commenced a phase II study of infusional fluorouracil. New drug combinations are also in development. One drug combination recently evaluated – VIG (vinblastine, ifosfamide and gallium) – was not recommended for further study. Other combinations of the newer drugs are at present under evaluation and randomized trial comparisons of these approaches are under way or at the planning stage. The experience of this study, however, does suggest that a word of caution is appropriate. It seems inherently unlikely that these new combinations will markedly increase the proportion of patients who are long-term survivors.

New prospective studies should therefore examine not only response rates and survival, but also quality of life to assess the true impact of therapies, which are often toxic, on this patient population.

In summary, this randomized trial demonstrated a clear improvement in symptomatic and objective progression-free survival, together with survival as a result of the addition of cisplatin to methotrexate and vinblastine. This improvement in anti-cancer effect was, however, achieved at the cost of increased toxicity. Cisplatin containing combinations can be recommended for patients in whom benefit is likely to exceed toxicity. New treatment approaches should be supported and evaluated in randomized clinical trials.

### ACKNOWLEDGEMENTS

We would like to thank Angela Crook and Andrea Bailey, who gave valuable statistical advice and input through the course of the trial.

Clinicians who entered patients into this trial: VL Barley, A Barrett, K Benstead, J Bolger, JM Bozzino, JES Brock, RL Canney, AE Champion, P Clark, MA Coe, R Cowan, SM Crawford, RD Errington, J Glaholm, JD Graham, T Habeshaw, RR Hall, SJ Harland, AN Harnett, PG Harper, ND James, R Jones, EJ Junor, AV Kaisary, DJ Kerr, B McIllmurray, M Mason, GM Mead, S Myint, HFV Newman, J Owen, R Rampling, JT Roberts, AG Robertson, JM Russell, AJ Slater, DB Smith, M Snee, W Steward, P Symonds, H Yosef.

### REFERENCES

Ahmed T, Yagoda A, Needles B, Scher HI, Watson RC and Geller N (1985) Vinblastine and methotrexate for advanced bladder cancer. *J Urol* **133**: 602–604

Cancer Research Campaign (1995) *Mortality-UK*. Factsheet 3. Cancer Research Campaign

Cockroft DW and Gault MH (1976) Prediction of creatinine clearance from serum creatinine. *Nephrology* **16**: 31–41

Fossa SD, Sternberg C, Scher HI, Theodore CH, Mead B, Dearnaley D, Roberts JT and Skovlund E (1996) Survival of patients with advanced urothelial cancer treated with cisplatin-based chemotherapy. *Br J Cancer* **74**: 1655–1659

Gagliano R, Levin H, El-Bolkainy MN, Wilson HE, Stephens RL, Fletcher WS, Rivkin SE, O'Bryan RM, Coltman CA, Saiki JH, Stuckey WJ, Balducci L, Bonnet JS and Nixon DO (1983) Adriamycin versus adriamycin plus cis-diamminedichloro-platinum (DDP) in advanced transitional cell bladder carcinoma. *Am J Clin Oncol* **6**: 215–218

Geller NL, Sternberg CN, Penenberg D, Scher H and Yagoda A (1991) Prognostic factors of survival of patients with advanced urothelial tumours treated with methotrexate, vinblastine, doxorubicin, and cisplatin chemotherapy. *Cancer* **67**: 1525–1531

Harker GW, Meyers FJ, Freiha FS, Palmer JM, Shortliffe LD, Hannigan JF, McWhirter KM and Torti FM (1985) Cisplatin, methotrexate and vinblastine (CMV): an effective chemotherapy regimen for metastatic transitional cell carcinoma of the urinary tract. A Northern Oncology Group Study. *J Clin Oncol* **31**: 463–470

Hillcoat BL, Raghaven D, Matthews J, Kefford R, Yuen K, Woods R, Olver I, Bishop J, Pearson B, Coorey G, Levi J, Abbott RL, Aroney R, Gill PG and McLennan R (1989) Cisplatin, methotrexate, and vinblastine (CMV): an effective chemotherapy regimen for metastatic transitional cell carcinoma of the urinary tract. A Northern California Oncology Group Study. *J Clin Oncol* **7**: 706–709

Jeffery GM and Mead GM (1992) CMV chemotherapy for advanced transitional cell carcinoma. *Br J Cancer* **66**: 542–546

Khandekar JD, Elson PJ, DeWys WD, Slayton RE and Harris DT (1985) Comparative activity and toxicity of cis-diamminedichloroplatinum (DDP) and a combination of doxorubicin, cyclophosphamide, and DDP in disseminated transitional cell carcinomas of the urinary tract. *J Clin Oncol* **3**: 539–545

Loehrer PJ, Einhorn LH, Elson PJ, Crawford ED, Kuebler P, Tannock I, Raghavan D, Stuart-Harris R, Sarosdy MF, Lowe BA, Lowe A, Blumenstein B and Trump D (1992) A randomised comparison of cisplatin alone or in combination with methotrexate, vinblastine, and doxorubicin in patients with metastatic urothelial carcinoma: a cooperative group study. *J Clin Oncol* **10**: 1066–1073

Loehrer PJ, Elson P, Dreicer R, Hahan R, Nichols CR, Williams R and Einhorn LH (1994) Escalated dosages of methotrexate, vinblastine, doxorubicin, and cisplatin plus recombinant human granulocyte colon-stimulating factor in advanced urothelial carcinoma: an Eastern Cooperative Oncology Group Trial. *J Clin Oncol* **12**: 483–488

Logothetis CJ, Dexeus FH, Finn L, Sella A, Amato RJ, Ayalo A G and Kilbourn RG (1990) A prospective randomised trial comparing MVAC and CISCA chemotherapy for patients with metastatic urothelial tumours. *J Clin Oncol* **8**: 1050–1055

Logothetis CJ, Finn LD, Smith T, Kilbourn RG, Ellerhorst JA, Zukiwski AA, Sella A, Tu SM and Amato RJ (1995) Escalated MVAC with or without recombinant human granulocyte–macrophage colony-stimulating factor for the initial treatment of advanced malignant urothelial tumours: results of a randomised trial. *J Clin Oncol* **13**: 2272–2277

McCaffrey L, Hilton S, Bajorin D, Maxumdar M, Amsterdam A, Kim B and Scher H (1995) Docetaxel in patients with advanced transitional cell cancer (TCC) who failed cisplatin-based chemotherapy: a phase II trial. *Proc ASCO* **14**: 233

Parmar MKB and Machin D (1995). *Survival Analysis: a Practical Approach*. John Wiley & Sons, Chichester, UK

Roth B J, Dreicer R, Einhorn LH, Neuberg D, Johnson DH, Smith JL, Hudes GR, Schultz SM and Loehrer PJ (1994) Significant activity of paclitaxel in advanced transitional-cell carcinoma of the urothelium: a phase II trial of the Eastern Cooperative Oncology Group. *J Clin Oncol* **12**: 2264–2270

SAS (1989) SAS Institute, Cary, N C, USA

Saxman SB, Propert KJ, Einhorn LH, Crawford ED, Tannock I, Raghavan D, Loehrer PJ and Trump D (1997) Long-term follow-up of a phase III inter-group study of cisplatin alone or in combination with methotrexate, vinblastine and doxorubicin in patients with metastatic urothelial carcinoma: a cooperative group study. *J Clin Oncol* **15**: 2564–2569

Seligman PA and Crawford E (1991) Treatment of advanced transitional cell carcinoma of the bladder with continuous-infusion gallium nitrate. *J Natl Cancer Inst* **83**: 1582–1584

Soloway MS, Einstein A, Corder MP, Bonney W, Prout GR and Coombs J (1983) A comparison of cisplatin and the combination of cisplatin and cyclophosphamide in advanced urothelial cancer. *Cancer* **52**: 767–772

Stadler W, Kuzel T, Raghavan D, Levine E, Vogelzang N and Dorr FA (1995) A phase II study of gemcitabine in the treatment of patients with advanced transitional cell carcinoma. *Proc ASCO* **14**: 241

 © Cancer Research Campaign 1998

Sternberg CN, Yagoda A, Scher HI, Watson RC, Ahmed T, Weiselberg LR, Geller N, Hollander PS, Herr HW, Sogani PC, Morse MJ and Whitmore WF (1985) Preliminary results of M-VAC (methotrexate, vinblastine, doxorubicin and cisplatin) for transitional cell carcinoma of the urothelium. *J Urol* **133**: 403–407

Sternberg CN, Yagoda A, Scher HI, Watson RC, Herr HW, Morse MJ, Sogani PC, Darracott Vaughan E, Bander N, Weiselberg LR, Geller N, Hollander PS, Lipperman R, Fair WR and Whitmore WF (1988) M-VAC (methotrexate, vinblastine, doxorubicin and cisplatin) for advanced transitional cell carcinoma of the urothelium. *J Urol* **139**: 461–469

Sternberg CR, Yagoda A, Scher HI, Watson RC, Geller N, Herr HW, Morse MJ, Sogani PC, Darracott Vaughan E, Bander N, Weiselberg L, Rosado K, Smart T, Lin S-Y, Penenberg D, Fair WR and Whitmore WF (1989) Methotrexate, vinblastine, doxorubicin and cisplatin for advanced transitional cell carcinoma of the urothelium. *Cancer* **64**: 2448–2458

Sternberg CN (1995) The treatment of advanced bladder cancer. *Ann Oncol* **6**: 113–126

Sternberg CN, De Mulder P, Fossa S, Schornagel J, Collette L and de Balincourt C (1997) Interim toxicity analysis of a randomised trial in advanced urothelial tract tumours of high-dose intensity MVAC chemotherapy (HD-MVAC) and recombinant human granulocyte colony-stimulating factor (G-CSF) versus classic MVAC chemotherapy. *Proc ASCO* **16**: 320

Tannock I, Gospodarowicz M, Connolly J and Jewett M (1989) M-VAC (methotrexate, vinblastine, doxorubicin and cisplatin) chemotherapy for transitional cell carcinoma: The Princess Margaret Hospital experience. *J Urol* **142**: 289–292

Troner M, Birch R, Omura GA and Williams S (1987) Phase II comparison of cisplatin alone versus cisplatin, doxorubicin and cyclophosphamide in the treatment of bladder (urothelial) cancer: a Southeastern Cancer Study Group Trial. *J Urol* **137**: 660–662

Witte RS, Elson P, Bono B, Knop R, Richardson RR, Dreicer R and Loehrer PJ (1997) Eastern Cooperative Oncology Group phase II trial of ifosfamide in the treatment of previously treated advanced urothelial carcinoma. *J Clin Oncol* **15**: 584–593

© Cancer Research Campaign 1998


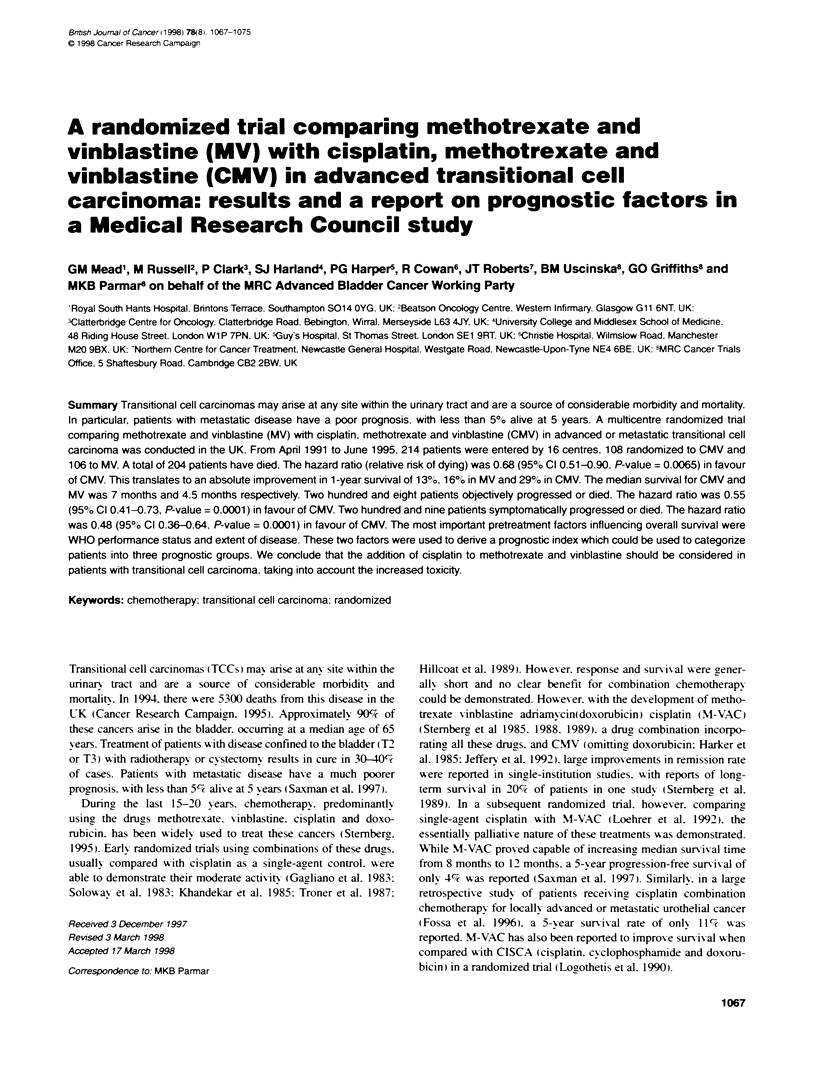

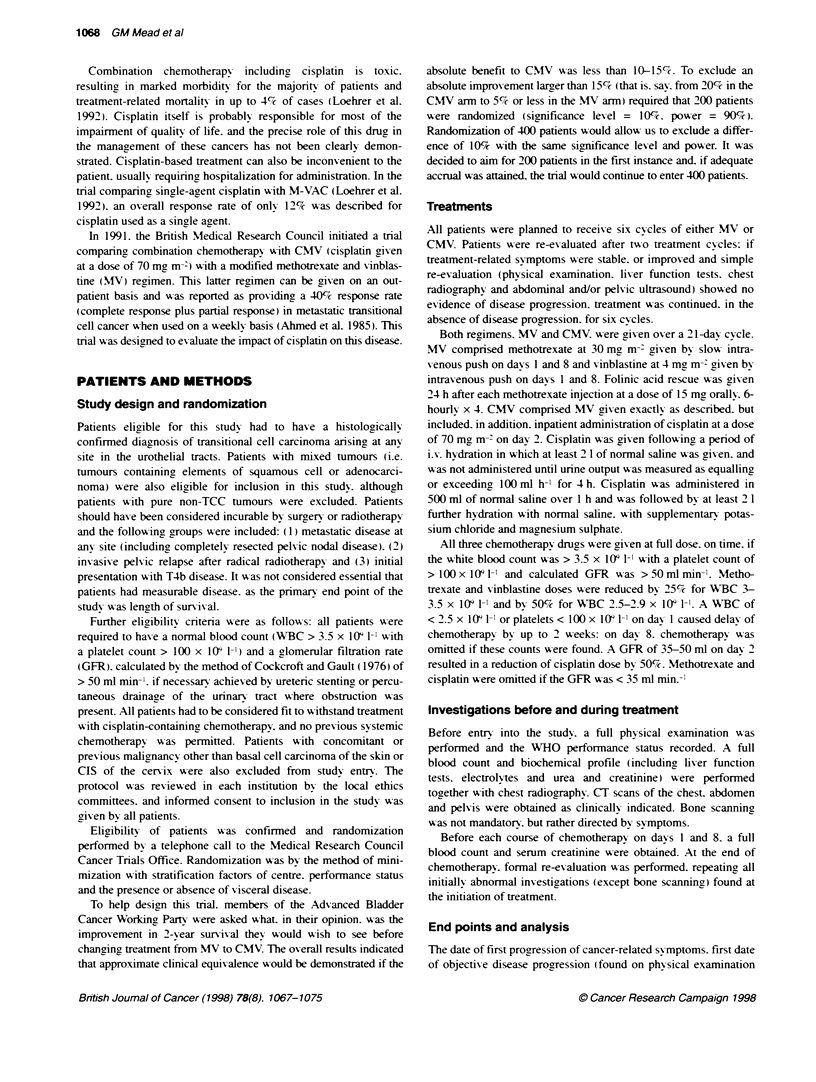

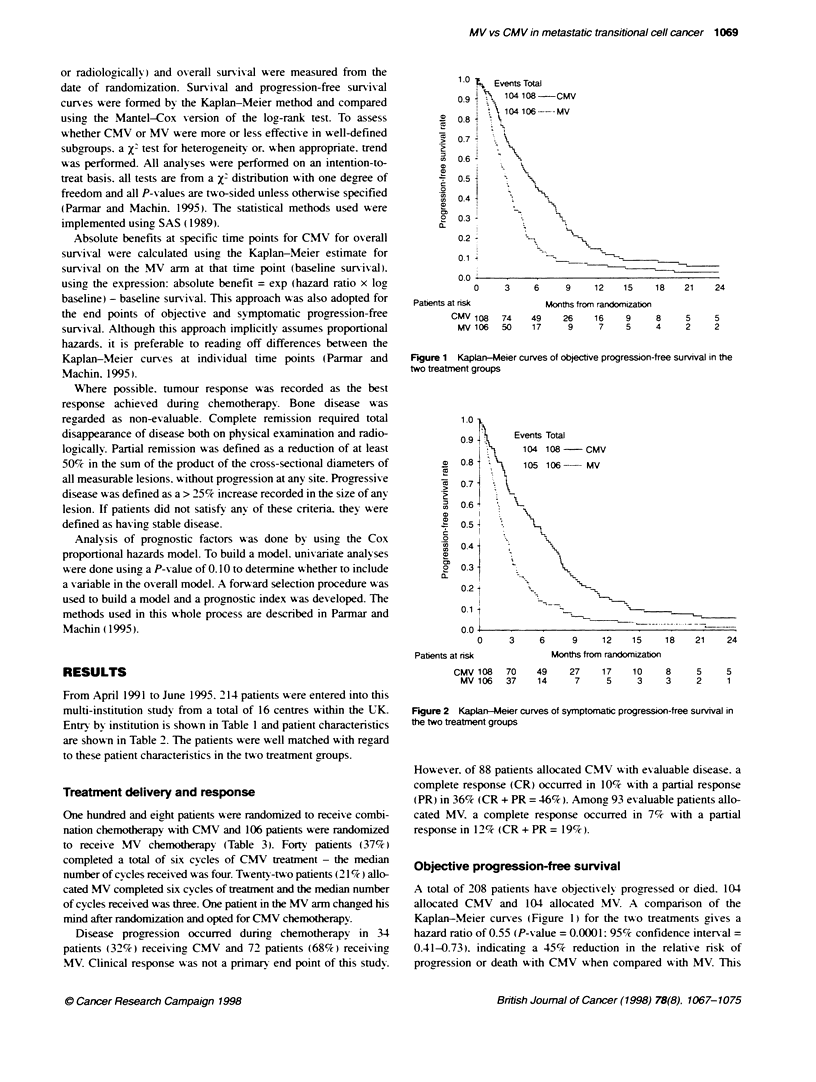

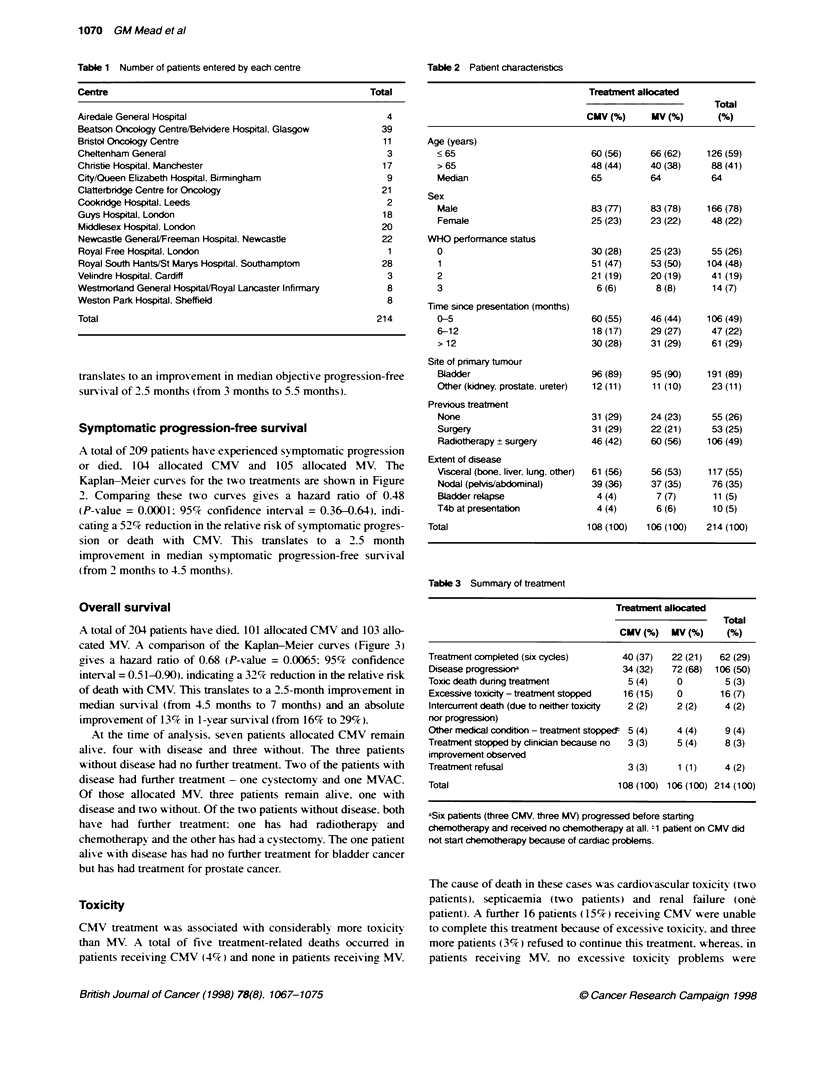

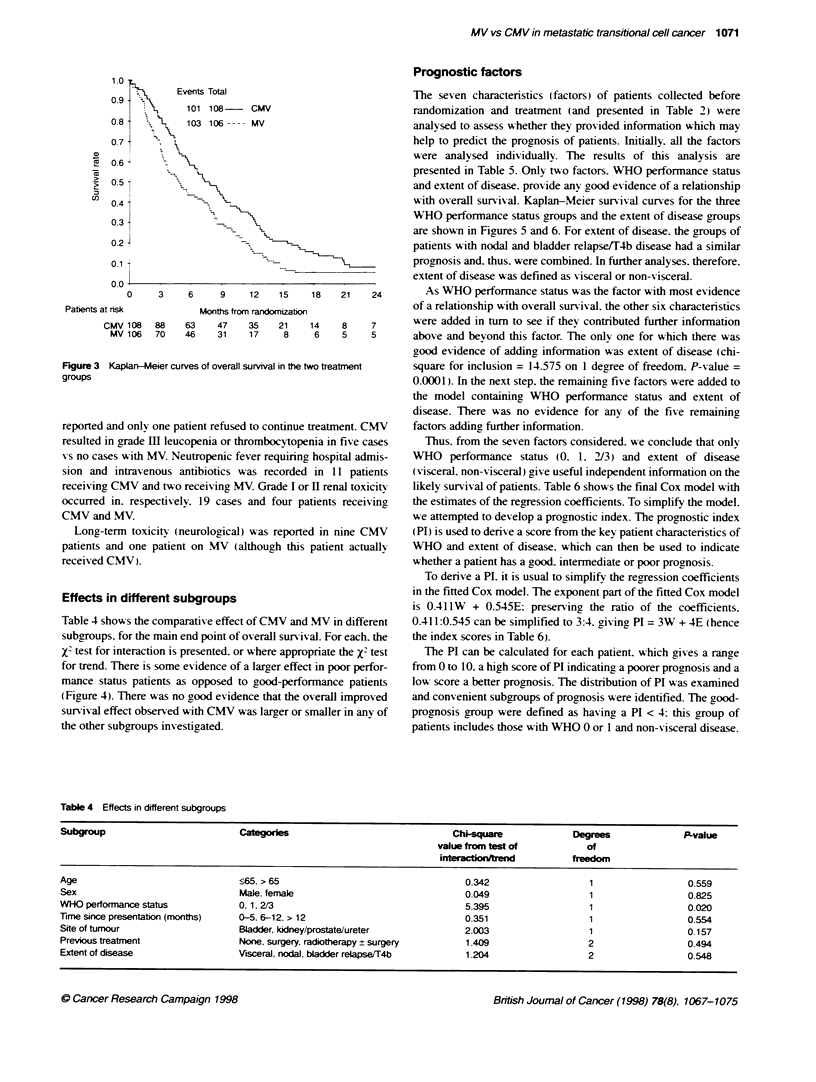

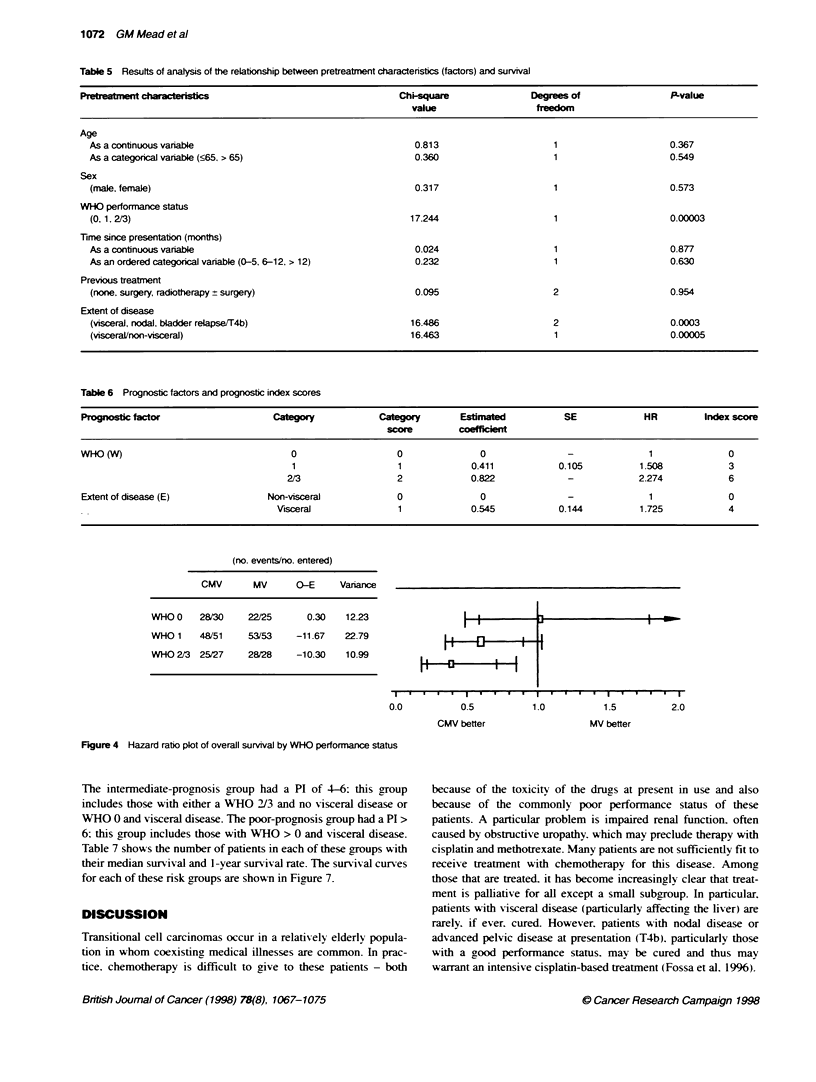

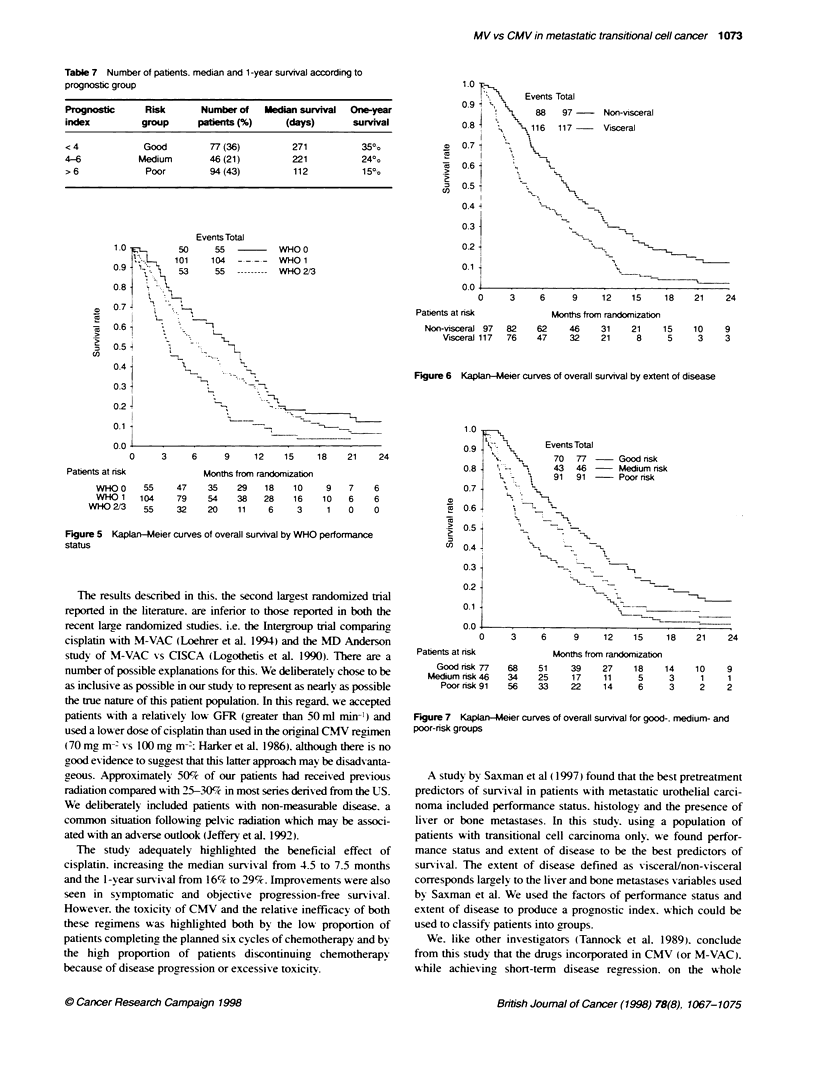

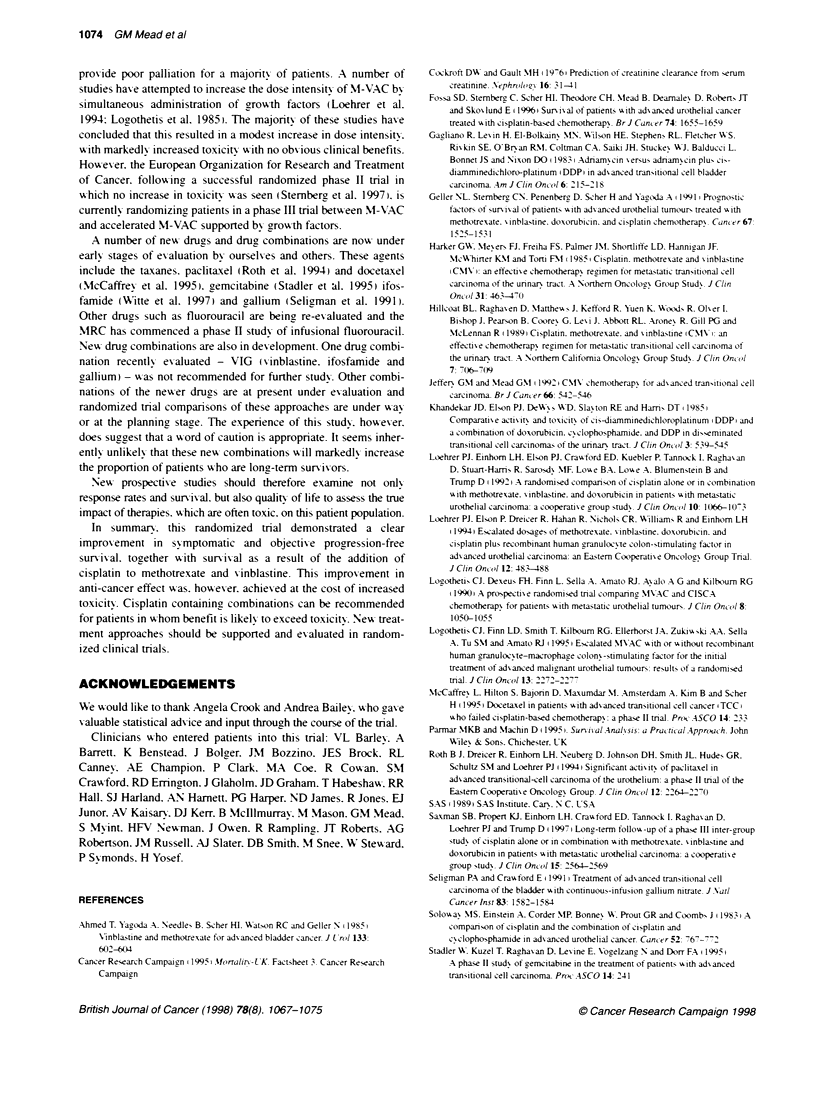

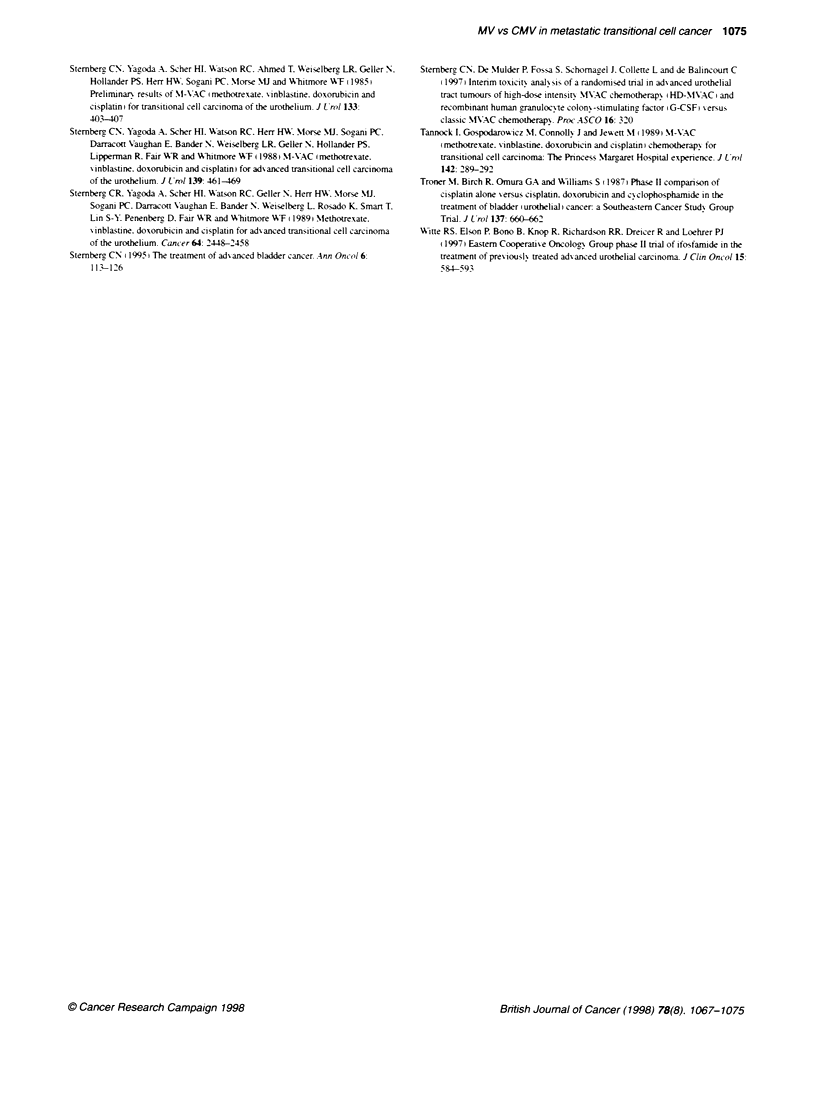


## References

[OCR_01149] Ahmed T., Yagoda A., Needles B., Scher H. I., Watson R. C., Geller N. (1985). Vinblastine and methotrexate for advanced bladder cancer.. J Urol.

[OCR_01156] Cockcroft D. W., Gault M. H. (1976). Prediction of creatinine clearance from serum creatinine.. Nephron.

[OCR_01160] Fosså S. D., Sternberg C., Scher H. I., Theodore C. H., Mead B., Dearnaley D., Roberts J. T., Skovlund E. (1996). Survival of patients with advanced urothelial cancer treated with cisplatin-based chemotherapy.. Br J Cancer.

[OCR_01201] Jeffery G. M., Mead G. M. (1992). CMV chemotherapy for advanced transitional cell carcinoma.. Br J Cancer.

[OCR_01209] Loehrer P. J., Einhorn L. H., Elson P. J., Crawford E. D., Kuebler P., Tannock I., Raghavan D., Stuart-Harris R., Sarosdy M. F., Lowe B. A. (1992). A randomized comparison of cisplatin alone or in combination with methotrexate, vinblastine, and doxorubicin in patients with metastatic urothelial carcinoma: a cooperative group study.. J Clin Oncol.

[OCR_01224] Logothetis C. J., Dexeus F. H., Finn L., Sella A., Amato R. J., Ayala A. G., Kilbourn R. G. (1990). A prospective randomized trial comparing MVAC and CISCA chemotherapy for patients with metastatic urothelial tumors.. J Clin Oncol.

[OCR_01250] Roth B. J., Dreicer R., Einhorn L. H., Neuberg D., Johnson D. H., Smith J. L., Hudes G. R., Schultz S. M., Loehrer P. J. (1994). Significant activity of paclitaxel in advanced transitional-cell carcinoma of the urothelium: a phase II trial of the Eastern Cooperative Oncology Group.. J Clin Oncol.

[OCR_01254] Saxman S. B., Propert K. J., Einhorn L. H., Crawford E. D., Tannock I., Raghavan D., Loehrer P. J., Trump D. (1997). Long-term follow-up of a phase III intergroup study of cisplatin alone or in combination with methotrexate, vinblastine, and doxorubicin in patients with metastatic urothelial carcinoma: a cooperative group study.. J Clin Oncol.

[OCR_01261] Seligman P. A., Crawford E. D. (1991). Treatment of advanced transitional cell carcinoma of the bladder with continuous-infusion gallium nitrate.. J Natl Cancer Inst.

[OCR_01268] Soloway M. S., Einstein A., Corder M. P., Bonney W., Prout G. R., Coombs J. (1983). A comparison of cisplatin and the combination of cisplatin and cyclophosphamide in advanced urothelial cancer. A National Bladder Cancer Collaborative Group A Study.. Cancer.

[OCR_01299] Sternberg C. N., Yagoda A., Scher H. I., Watson R. C., Geller N., Herr H. W., Morse M. J., Sogani P. C., Vaughan E. D., Bander N. (1989). Methotrexate, vinblastine, doxorubicin, and cisplatin for advanced transitional cell carcinoma of the urothelium. Efficacy and patterns of response and relapse.. Cancer.

[OCR_01289] Sternberg C. N., Yagoda A., Scher H. I., Watson R. C., Herr H. W., Morse M. J., Sogani P. C., Vaughan E. D., Bander N., Weiselberg L. R. (1988). M-VAC (methotrexate, vinblastine, doxorubicin and cisplatin) for advanced transitional cell carcinoma of the urothelium.. J Urol.

[OCR_01316] Tannock I., Gospodarowicz M., Connolly J., Jewett M. (1989). M-VAC (methotrexate, vinblastine, doxorubicin and cisplatin) chemotherapy for transitional cell carcinoma: the Princess Margaret Hospital experience.. J Urol.

[OCR_01323] Troner M., Birch R., Omura G. A., Williams S. (1987). Phase III comparison of cisplatin alone versus cisplatin, doxorubicin and cyclophosphamide in the treatment of bladder (urothelial) cancer: a Southeastern Cancer Study Group trial.. J Urol.

[OCR_01330] Witte R. S., Elson P., Bono B., Knop R., Richardson R. R., Dreicer R., Loehrer P. J. (1997). Eastern Cooperative Oncology Group phase II trial of ifosfamide in the treatment of previously treated advanced urothelial carcinoma.. J Clin Oncol.

